# (*E*)-*N*′-(3-Benz­yloxy-4-methoxy­benzyl­idene)isonicotinohydrazide

**DOI:** 10.1107/S1600536809037921

**Published:** 2009-09-26

**Authors:** H. S. Naveenkumar, Amirin Sadikun, Pazilah Ibrahim, Wan-Sin Loh, Hoong-Kun Fun

**Affiliations:** a School of Pharmaceutical Sciences, Universiti Sains Malaysia, 11800 USM, Penang, Malaysia; b X-ray Crystallography Unit, School of Physics, Universiti Sains Malaysia, 11800 USM, Penang, Malaysia

## Abstract

In the title compound, C_21_H_19_N_3_O_3_, the pyridine ring forms a dihedral angle of 15.25 (6)° with the benzene ring. The dihedral angle between the two benzene rings is 83.66 (7)°. The meth­oxy group is slightly twisted away from the attached ring [C—O—C—C = 7.5 (2)°]. In the crystal structure, mol­ecules are linked into a three-dimensional network by inter­molecular N—H⋯N and C—H⋯O hydrogen bonds. The structure is further stabilized by C—H⋯π inter­actions.

## Related literature

For bond-length data, see: Allen *et al.* (1987 ▶). For applications of isoniazid derivatives, see: Janin (2007 ▶); Maccari *et al.* (2005 ▶); Slayden & Barry (2000 ▶). For the preparation, see: Lourenço *et al.* (2008 ▶). For the biological activity of Schiff bases, see: Kahwa *et al.* (1986 ▶). For related structures, see: Naveenkumar, Sadikun, Ibrahim, Goh & Fun (2009 ▶); Naveenkumar, Sadikun, Ibrahim, Yeap & Fun (2009 ▶); Shi (2005 ▶). For the stability of the temperature controller used for the data collection, see: Cosier & Glazer (1986 ▶).
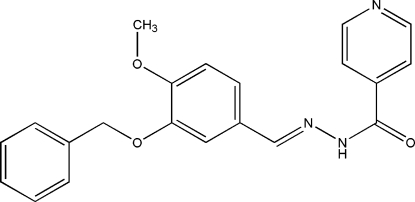


## Experimental

### 

#### Crystal data

C_21_H_19_N_3_O_3_
*M* *_r_* = 361.39
Monoclinic, 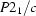

*a* = 18.3930 (6) Å
*b* = 11.5574 (4) Å
*c* = 8.3508 (3) Å
β = 93.436 (2)°
*V* = 1771.98 (11) Å^3^
*Z* = 4
Mo *K*α radiation
μ = 0.09 mm^−1^
*T* = 100 K
0.71 × 0.13 × 0.09 mm

#### Data collection

Bruker SMART APEXII CCD area-detector diffractometer
Absorption correction: multi-scan (**SADABS**; Bruker, 2005 ▶) *T*_min_ = 0.937, *T*_max_ = 0.992
27863 measured reflections
6434 independent reflections
3841 reflections with *I* > 2σ(*I*)
*R*_int_ = 0.061

#### Refinement

*R*[*F* ^2^ > 2σ(*F* ^2^)] = 0.060
*wR*(*F* ^2^) = 0.145
*S* = 1.06
6433 reflections
249 parameters
H atoms treated by a mixture of independent and constrained refinement
Δρ_max_ = 0.37 e Å^−3^
Δρ_min_ = −0.28 e Å^−3^

### 

Data collection: *APEX2* (Bruker, 2005 ▶); cell refinement: *SAINT* (Bruker, 2005 ▶); data reduction: *SAINT*; program(s) used to solve structure: *SHELXTL* (Sheldrick, 2008 ▶); program(s) used to refine structure: *SHELXTL*; molecular graphics: *SHELXTL*; software used to prepare material for publication: *SHELXTL* and *PLATON* (Spek, 2009 ▶).

## Supplementary Material

Crystal structure: contains datablocks global, I. DOI: 10.1107/S1600536809037921/ci2917sup1.cif

Structure factors: contains datablocks I. DOI: 10.1107/S1600536809037921/ci2917Isup2.hkl

Additional supplementary materials:  crystallographic information; 3D view; checkCIF report

## Figures and Tables

**Table 1:** Hydrogen-bond geometry (Å, °) *Cg*1 is the centroid of the C8–C13 ring.

*D*—H⋯*A*	*D*—H	H⋯*A*	*D*⋯*A*	*D*—H⋯*A*
N2—H1N2⋯N1^i^	0.88 (2)	2.54 (2)	3.3122 (17)	146 (1)
C9—H9*A*⋯O1^ii^	0.93	2.55	3.3524 (17)	144
C19—H19*A*⋯O3^iii^	0.93	2.54	3.3960 (17)	153
C17—H17*A*⋯*Cg*1^iv^	0.93	2.93	3.6694 (17)	137

Symmetry codes: (i) 
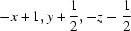
; (ii) 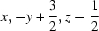; (iii) 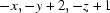; (iv) 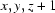.

**Fig. 1.**
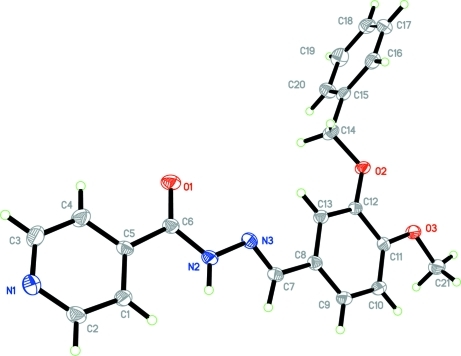
The molecular structure of the title compound, showing 50% probability displacement ellipsoids and the atom-numbering scheme.

**Fig. 2.**
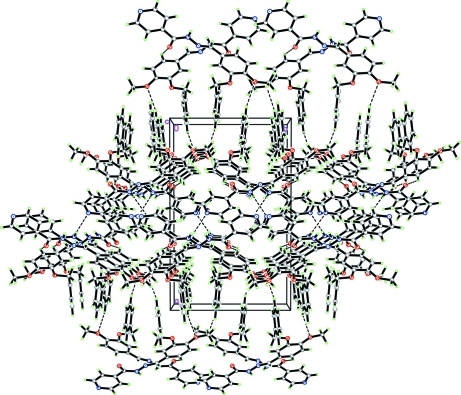
The crystal packing of the title compound, viewed along the c axis. Hydrogen bonds are shown as dashed lines.

## supplementary crystallographic information

### Comment

In the search of new compounds, isoniazid derivatives have been found to possess potential tuberculostatic activity (Janin, 2007; Maccari *et al.*, 2005; Slayden & Barry, 2000). Schiff bases have attracted much attention because of their biological activity (Kahwa *et al.*, 1986). As a part of a current work of synthesis of (*E*)-*N*'-(substituted-benzylidene) isonicotinohydrazide derivatives, in this paper we present the crystal structure of the title compound.

Bond lengths (Allen *et al.*, 1987) and the angles of the title compound (Fig. 1) are within the normal range and are comparable to those observed for closely related structures (Naveenkumar, Sadikun, Ibrahim, Goh & Fun, 2009; Naveenkumar, Sadikun, Ibrahim, Yeap & Fun, 2009). The mean plane of pyridine (C1–C5/N1) ring forms a dihedral angle of 15.25 (6)° with the benzene (C8–C13) ring. The two benzene (C8–C13 and C15–C20) rings form a dihedral angle of 83.66 (7)° with each other.

In the crystal packing (Fig. 2), molecules are linked into a three-dimensional network by intermolecular N2—H1N2···N1, C9—H9A···O1 and C19—H19A···O3 hydrogen bonds. The crystal structure is further stabilized by C17—H17A···*Cg*1 interactions (Table 1; *Cg*1 is the centroid of the C8-C13 benzene ring).

### Experimental

The isoniazid (INH) derivative was prepared following the procedure by literature (Lourenço *et al.*, 2008). (*E*)—*N*'-(3-Benzyloxy-4-methoxybenzylidene)isonicotinohydrazide was prepared by reaction between the 3-benzyloxy-4-methoxy benzaldehyde (1.0 eq) with INH (1.0 eq) in ethanol/water (10 ml), initially dissolving the INH in water and adding the respective solution over a solution of the aldehyde in ethanol. After stirring for 1 to 3 h at room temperature, the resulting mixture was concentrated under reduced pressure. The residue was purified by washing with cold ethyl alcohol and ethyl ether to afford the pure derivative. Yellow single crystals suitable for X-ray analysis were obtained by slow evaporation of a dimethyl sulfoxide solution at room temperature.

### Refinement

All carbon-bound H atoms were positioned geometrically [C–H = 0.93-0.97 Å] and were refined using a riding model, with *U*_iso_(H) = 1.2 or 1.5 *U*_eq_(C). A rotating-group model was applied for the methyl group. Atom H1N2 was located in a difference Fourier map and refined freely.

### Crystal data

**Table:**

C_21_H_19_N_3_O_3_	*F*(000) = 760
*M**_r_* = 361.39	*D*_x_ = 1.355 Mg m^−^^3^
Monoclinic, *P*2_1_/*c*	Mo *K*α radiation, λ = 0.71073 Å
Hall symbol: -P 2ybc	Cell parameters from 4601 reflections
*a* = 18.3930 (6) Å	θ = 2.8–32.1°
*b* = 11.5574 (4) Å	µ = 0.09 mm^−^^1^
*c* = 8.3508 (3) Å	*T* = 100 K
β = 93.436 (2)°	Needle, yellow
*V* = 1771.98 (11) Å^3^	0.71 × 0.13 × 0.09 mm
*Z* = 4	

### Data collection

**Table:**

Bruker SMART APEXII CCD area-detector diffractometer	6434 independent reflections
Radiation source: fine-focus sealed tube	3841 reflections with *I* > 2σ(*I*)
graphite	*R*_int_ = 0.061
φ and ω scans	θ_max_ = 32.6°, θ_min_ = 1.1°
Absorption correction: multi-scan (*SADABS*; Bruker, 2005)	*h* = −26→27
*T*_min_ = 0.937, *T*_max_ = 0.992	*k* = −15→17
27863 measured reflections	*l* = −12→12

### Refinement

**Table:**

Refinement on *F*^2^	Primary atom site location: structure-invariant direct methods
Least-squares matrix: full	Secondary atom site location: difference Fourier map
*R*[*F*^2^ > 2σ(*F*^2^)] = 0.060	Hydrogen site location: inferred from neighbouring sites
*wR*(*F*^2^) = 0.145	H atoms treated by a mixture of independent and constrained refinement
*S* = 1.06	*w* = 1/[σ^2^(*F*_o_^2^) + (0.0618*P*)^2^] where *P* = (*F*_o_^2^ + 2*F*_c_^2^)/3
6433 reflections	(Δ/σ)_max_ = 0.001
249 parameters	Δρ_max_ = 0.37 e Å^−^^3^
0 restraints	Δρ_min_ = −0.28 e Å^−^^3^

### Special details

**Table:**

Experimental. The crystal was placed in the cold stream of an Oxford Cyrosystems Cobra open-flow nitrogen cryostat (Cosier & Glazer, 1986) operating at 100.0 (1) K.
Geometry. All esds (except the esd in the dihedral angle between two l.s. planes) are estimated using the full covariance matrix. The cell esds are taken into account individually in the estimation of esds in distances, angles and torsion angles; correlations between esds in cell parameters are only used when they are defined by crystal symmetry. An approximate (isotropic) treatment of cell esds is used for estimating esds involving l.s. planes.
Refinement. Refinement of *F*^2^ against ALL reflections. The weighted *R*-factor *wR* and goodness of fit *S* are based on *F*^2^, conventional *R*-factors *R* are based on *F*, with *F* set to zero for negative *F*^2^. The threshold expression of *F*^2^ > σ(*F*^2^) is used only for calculating *R*-factors(gt) *etc*. and is not relevant to the choice of reflections for refinement. *R*-factors based on *F*^2^ are statistically about twice as large as those based on *F*, and *R*- factors based on ALL data will be even larger.

### Fractional atomic coordinates and isotropic or equivalent isotropic displacement parameters (Å^2^)

**Table:**

	*x*	*y*	*z*	*U*_iso_*/*U*_eq_	
O1	0.34254 (5)	0.51645 (9)	0.11366 (12)	0.0270 (2)	
O2	0.17576 (5)	0.99561 (8)	0.38579 (11)	0.0206 (2)	
O3	0.17536 (5)	1.19805 (8)	0.25594 (11)	0.0221 (2)	
N1	0.49029 (7)	0.30387 (11)	−0.27715 (14)	0.0258 (3)	
N2	0.38144 (6)	0.66534 (10)	−0.03723 (14)	0.0205 (3)	
N3	0.34440 (6)	0.74619 (10)	0.04977 (13)	0.0205 (3)	
C1	0.46166 (7)	0.50212 (12)	−0.22030 (17)	0.0228 (3)	
H1A	0.4687	0.5799	−0.2432	0.027*	
C2	0.49753 (8)	0.41733 (13)	−0.30194 (17)	0.0239 (3)	
H2A	0.5287	0.4409	−0.3792	0.029*	
C3	0.44347 (8)	0.27391 (13)	−0.16720 (19)	0.0310 (4)	
H3A	0.4361	0.1955	−0.1492	0.037*	
C4	0.40545 (8)	0.35211 (13)	−0.07883 (18)	0.0265 (3)	
H4A	0.3738	0.3263	−0.0037	0.032*	
C5	0.41511 (7)	0.46942 (12)	−0.10385 (15)	0.0182 (3)	
C6	0.37626 (7)	0.55180 (12)	0.00140 (16)	0.0190 (3)	
C7	0.33453 (7)	0.84479 (12)	−0.02067 (16)	0.0192 (3)	
H7A	0.3528	0.8565	−0.1209	0.023*	
C8	0.29549 (7)	0.93788 (12)	0.05350 (16)	0.0185 (3)	
C9	0.29436 (7)	1.04692 (12)	−0.01554 (16)	0.0205 (3)	
H9A	0.3198	1.0602	−0.1068	0.025*	
C10	0.25541 (7)	1.13699 (12)	0.05066 (16)	0.0211 (3)	
H10A	0.2556	1.2102	0.0044	0.025*	
C11	0.21677 (7)	1.11759 (11)	0.18421 (16)	0.0182 (3)	
C12	0.21699 (7)	1.00623 (11)	0.25633 (15)	0.0173 (3)	
C13	0.25588 (7)	0.91782 (12)	0.18985 (16)	0.0186 (3)	
H13A	0.2559	0.8445	0.2356	0.022*	
C14	0.16762 (8)	0.88010 (12)	0.44760 (18)	0.0246 (3)	
H14A	0.2148	0.8492	0.4839	0.030*	
H14B	0.1466	0.8299	0.3641	0.030*	
C15	0.11900 (7)	0.88528 (11)	0.58412 (17)	0.0197 (3)	
C16	0.14817 (8)	0.90316 (12)	0.73981 (18)	0.0255 (3)	
H16A	0.1983	0.9111	0.7585	0.031*	
C17	0.10355 (9)	0.90916 (13)	0.86672 (18)	0.0312 (4)	
H17A	0.1236	0.9215	0.9702	0.037*	
C18	0.02901 (9)	0.89689 (13)	0.83993 (19)	0.0312 (4)	
H18A	−0.0011	0.9007	0.9254	0.037*	
C19	−0.00072 (8)	0.87894 (13)	0.6859 (2)	0.0318 (4)	
H19A	−0.0508	0.8707	0.6677	0.038*	
C20	0.04401 (8)	0.87320 (13)	0.55915 (18)	0.0260 (3)	
H20A	0.0237	0.8611	0.4558	0.031*	
C21	0.16494 (9)	1.30698 (12)	0.17427 (18)	0.0280 (3)	
H21A	0.1321	1.3542	0.2310	0.042*	
H21B	0.1449	1.2936	0.0671	0.042*	
H21C	0.2109	1.3459	0.1703	0.042*	
H1N2	0.4037 (9)	0.6892 (16)	−0.122 (2)	0.046 (5)*	

### Atomic displacement parameters (Å^2^)

**Table:**

	*U*^11^	*U*^22^	*U*^33^	*U*^12^	*U*^13^	*U*^23^
O1	0.0320 (5)	0.0276 (6)	0.0229 (5)	0.0005 (5)	0.0133 (5)	0.0027 (4)
O2	0.0252 (5)	0.0174 (5)	0.0203 (5)	0.0004 (4)	0.0108 (4)	0.0016 (4)
O3	0.0297 (5)	0.0164 (5)	0.0210 (5)	0.0043 (4)	0.0079 (4)	0.0011 (4)
N1	0.0284 (6)	0.0237 (6)	0.0258 (6)	0.0024 (5)	0.0045 (5)	−0.0026 (5)
N2	0.0244 (6)	0.0200 (6)	0.0179 (6)	0.0025 (5)	0.0094 (5)	−0.0010 (5)
N3	0.0211 (6)	0.0210 (6)	0.0201 (6)	0.0020 (5)	0.0076 (5)	−0.0033 (5)
C1	0.0271 (7)	0.0185 (7)	0.0236 (7)	0.0026 (6)	0.0083 (6)	0.0020 (6)
C2	0.0248 (7)	0.0265 (8)	0.0212 (7)	0.0042 (6)	0.0084 (6)	0.0012 (6)
C3	0.0362 (8)	0.0188 (7)	0.0390 (9)	−0.0011 (7)	0.0123 (7)	0.0000 (6)
C4	0.0271 (7)	0.0220 (7)	0.0315 (8)	0.0000 (6)	0.0113 (6)	0.0032 (6)
C5	0.0181 (6)	0.0211 (7)	0.0156 (6)	0.0020 (5)	0.0036 (5)	0.0004 (5)
C6	0.0187 (6)	0.0204 (7)	0.0183 (7)	0.0007 (5)	0.0038 (5)	0.0006 (5)
C7	0.0183 (6)	0.0223 (7)	0.0178 (6)	−0.0007 (5)	0.0062 (5)	−0.0009 (5)
C8	0.0171 (6)	0.0206 (7)	0.0180 (6)	−0.0010 (5)	0.0034 (5)	−0.0020 (5)
C9	0.0217 (7)	0.0224 (7)	0.0180 (7)	−0.0013 (6)	0.0060 (5)	0.0009 (5)
C10	0.0247 (7)	0.0182 (7)	0.0207 (7)	−0.0007 (6)	0.0034 (6)	0.0023 (5)
C11	0.0200 (6)	0.0174 (6)	0.0175 (6)	0.0003 (5)	0.0027 (5)	−0.0021 (5)
C12	0.0172 (6)	0.0187 (6)	0.0163 (6)	−0.0020 (5)	0.0049 (5)	−0.0011 (5)
C13	0.0193 (6)	0.0178 (6)	0.0190 (7)	−0.0001 (5)	0.0043 (5)	0.0011 (5)
C14	0.0287 (7)	0.0166 (7)	0.0301 (8)	0.0018 (6)	0.0139 (6)	0.0041 (6)
C15	0.0236 (7)	0.0142 (6)	0.0221 (7)	0.0016 (5)	0.0084 (6)	0.0016 (5)
C16	0.0274 (7)	0.0196 (7)	0.0294 (8)	−0.0022 (6)	−0.0005 (6)	0.0015 (6)
C17	0.0507 (10)	0.0242 (8)	0.0188 (7)	0.0000 (7)	0.0031 (7)	−0.0011 (6)
C18	0.0440 (9)	0.0239 (8)	0.0280 (8)	0.0008 (7)	0.0208 (7)	0.0000 (6)
C19	0.0238 (7)	0.0328 (9)	0.0401 (10)	−0.0015 (6)	0.0126 (7)	−0.0020 (7)
C20	0.0251 (7)	0.0315 (8)	0.0217 (7)	0.0011 (6)	0.0048 (6)	−0.0015 (6)
C21	0.0405 (9)	0.0181 (7)	0.0260 (8)	0.0074 (6)	0.0074 (7)	0.0032 (6)

### Geometric parameters (Å, °)

**Table:**

O1—C6	1.2249 (16)	C9—C10	1.3964 (19)
O2—C12	1.3628 (16)	C9—H9A	0.93
O2—C14	1.4423 (16)	C10—C11	1.3763 (19)
O3—C11	1.3633 (16)	C10—H10A	0.93
O3—C21	1.4391 (16)	C11—C12	1.4208 (18)
N1—C2	1.3354 (19)	C12—C13	1.3826 (18)
N1—C3	1.3414 (19)	C13—H13A	0.93
N2—C6	1.3560 (18)	C14—C15	1.492 (2)
N2—N3	1.3871 (16)	C14—H14A	0.97
N2—H1N2	0.885 (18)	C14—H14B	0.97
N3—C7	1.2902 (17)	C15—C20	1.390 (2)
C1—C2	1.3836 (19)	C15—C16	1.392 (2)
C1—C5	1.3861 (19)	C16—C17	1.380 (2)
C1—H1A	0.93	C16—H16A	0.93
C2—H2A	0.93	C17—C18	1.383 (2)
C3—C4	1.383 (2)	C17—H17A	0.93
C3—H3A	0.93	C18—C19	1.383 (2)
C4—C5	1.3849 (19)	C18—H18A	0.93
C4—H4A	0.93	C19—C20	1.380 (2)
C5—C6	1.5049 (19)	C19—H19A	0.93
C7—C8	1.4527 (19)	C20—H20A	0.93
C7—H7A	0.93	C21—H21A	0.96
C8—C9	1.3855 (19)	C21—H21B	0.96
C8—C13	1.4072 (18)	C21—H21C	0.96
			
C12—O2—C14	116.22 (10)	O3—C11—C12	114.78 (11)
C11—O3—C21	116.78 (10)	C10—C11—C12	120.17 (12)
C2—N1—C3	115.70 (13)	O2—C12—C13	125.30 (12)
C6—N2—N3	118.92 (12)	O2—C12—C11	115.49 (11)
C6—N2—H1N2	122.3 (12)	C13—C12—C11	119.18 (12)
N3—N2—H1N2	118.4 (12)	C12—C13—C8	120.56 (12)
C7—N3—N2	114.60 (11)	C12—C13—H13A	119.7
C2—C1—C5	119.03 (13)	C8—C13—H13A	119.7
C2—C1—H1A	120.5	O2—C14—C15	108.43 (11)
C5—C1—H1A	120.5	O2—C14—H14A	110.0
N1—C2—C1	124.35 (14)	C15—C14—H14A	110.0
N1—C2—H2A	117.8	O2—C14—H14B	110.0
C1—C2—H2A	117.8	C15—C14—H14B	110.0
N1—C3—C4	124.23 (14)	H14A—C14—H14B	108.4
N1—C3—H3A	117.9	C20—C15—C16	118.60 (13)
C4—C3—H3A	117.9	C20—C15—C14	121.09 (13)
C3—C4—C5	119.06 (14)	C16—C15—C14	120.30 (13)
C3—C4—H4A	120.5	C17—C16—C15	120.72 (14)
C5—C4—H4A	120.5	C17—C16—H16A	119.6
C4—C5—C1	117.58 (13)	C15—C16—H16A	119.6
C4—C5—C6	117.49 (12)	C16—C17—C18	119.99 (14)
C1—C5—C6	124.89 (13)	C16—C17—H17A	120.0
O1—C6—N2	123.42 (13)	C18—C17—H17A	120.0
O1—C6—C5	121.06 (13)	C19—C18—C17	119.91 (15)
N2—C6—C5	115.52 (12)	C19—C18—H18A	120.0
N3—C7—C8	121.29 (12)	C17—C18—H18A	120.0
N3—C7—H7A	119.4	C20—C19—C18	119.98 (14)
C8—C7—H7A	119.4	C20—C19—H19A	120.0
C9—C8—C13	119.41 (12)	C18—C19—H19A	120.0
C9—C8—C7	119.45 (12)	C19—C20—C15	120.79 (14)
C13—C8—C7	121.07 (12)	C19—C20—H20A	119.6
C8—C9—C10	120.56 (13)	C15—C20—H20A	119.6
C8—C9—H9A	119.7	O3—C21—H21A	109.5
C10—C9—H9A	119.7	O3—C21—H21B	109.5
C11—C10—C9	120.10 (13)	H21A—C21—H21B	109.5
C11—C10—H10A	120.0	O3—C21—H21C	109.5
C9—C10—H10A	120.0	H21A—C21—H21C	109.5
O3—C11—C10	125.04 (12)	H21B—C21—H21C	109.5
			
C6—N2—N3—C7	−160.87 (12)	C9—C10—C11—O3	−178.10 (12)
C3—N1—C2—C1	1.4 (2)	C9—C10—C11—C12	0.70 (19)
C5—C1—C2—N1	0.4 (2)	C14—O2—C12—C13	−5.78 (18)
C2—N1—C3—C4	−1.8 (2)	C14—O2—C12—C11	172.17 (11)
N1—C3—C4—C5	0.3 (2)	O3—C11—C12—O2	0.27 (16)
C3—C4—C5—C1	1.5 (2)	C10—C11—C12—O2	−178.65 (11)
C3—C4—C5—C6	−176.45 (12)	O3—C11—C12—C13	178.35 (11)
C2—C1—C5—C4	−1.84 (19)	C10—C11—C12—C13	−0.56 (19)
C2—C1—C5—C6	175.95 (12)	O2—C12—C13—C8	178.59 (12)
N3—N2—C6—O1	−2.77 (19)	C11—C12—C13—C8	0.71 (18)
N3—N2—C6—C5	177.17 (10)	C9—C8—C13—C12	−0.98 (19)
C4—C5—C6—O1	6.12 (19)	C7—C8—C13—C12	−177.89 (12)
C1—C5—C6—O1	−171.67 (13)	C12—O2—C14—C15	−178.00 (10)
C4—C5—C6—N2	−173.82 (12)	O2—C14—C15—C20	89.96 (15)
C1—C5—C6—N2	8.39 (18)	O2—C14—C15—C16	−89.64 (15)
N2—N3—C7—C8	178.85 (11)	C20—C15—C16—C17	−0.2 (2)
N3—C7—C8—C9	171.47 (12)	C14—C15—C16—C17	179.39 (13)
N3—C7—C8—C13	−11.63 (19)	C15—C16—C17—C18	0.3 (2)
C13—C8—C9—C10	1.11 (19)	C16—C17—C18—C19	−0.2 (2)
C7—C8—C9—C10	178.06 (12)	C17—C18—C19—C20	0.1 (2)
C8—C9—C10—C11	−0.98 (19)	C18—C19—C20—C15	0.0 (2)
C21—O3—C11—C10	7.50 (19)	C16—C15—C20—C19	0.1 (2)
C21—O3—C11—C12	−171.35 (11)	C14—C15—C20—C19	−179.52 (13)

### Hydrogen-bond geometry (Å, °)

**Table:**

*D*—H···*A*	*D*—H	H···*A*	*D*···*A*	*D*—H···*A*
N2—H1N2···N1^i^	0.88 (2)	2.54 (2)	3.3122 (17)	146 (1)
C9—H9A···O1^ii^	0.93	2.55	3.3524 (17)	144
C19—H19A···O3^iii^	0.93	2.54	3.3960 (17)	153
C17—H17A···Cg1^iv^	0.93	2.93	3.6694 (17)	137

Symmetry codes: (i) −*x*+1, *y*+1/2, −*z*−1/2; (ii) *x*, −*y*+3/2, *z*−1/2; (iii) −*x*, −*y*+2, −*z*+1; (iv) *x*, *y*, *z*+1.
